# Knockdown of PEX16 Induces Autophagic Degradation of Peroxisomes

**DOI:** 10.3390/ijms22157989

**Published:** 2021-07-26

**Authors:** Xiaofan Wei, Yunash Maharjan, Debra Dorotea, Raghbendra-Kumar Dutta, Donghyun Kim, Hyunsoo Kim, Yizhu Mu, Channy Park, Raekil Park

**Affiliations:** Department of Biomedical Science & Engineering, Gwangju Institute of Science and Technology, Gwangju 61005, Korea; weixiaofan1@gist.ac.kr (X.W.); yunas_rose@yahoo.com (Y.M.); flodebra@gist.ac.kr (D.D.); raghavdutta123@gist.ac.kr (R.-K.D.); paul1480@gist.ac.kr (D.K.); ruyhyunsookim@gist.ac.kr (H.K.); yizhumu1202@outlook.com (Y.M.); channy2002@gmail.com (C.P.)

**Keywords:** peroxisomes, peroxins, PEX16, autophagy, pexophagy, chloroquine

## Abstract

Peroxisome abundance is regulated by homeostasis between the peroxisomal biogenesis and degradation processes. Peroxin 16 (PEX16) is a peroxisomal protein involved in trafficking membrane proteins for de novo peroxisome biogenesis. The present study demonstrates that PEX16 also modulates peroxisome abundance through pexophagic degradation. PEX16 knockdown in human retinal pigment epithelial-1 cells decreased peroxisome abundance and function, represented by reductions in the expression of peroxisome membrane protein ABCD3 and the levels of cholesterol and plasmalogens, respectively. The activation of pexophagy under PEX16 knockdown was shown by (i) abrogated peroxisome loss under PEX16 knockdown in autophagy-deficient ATG5 knockout cell lines, and (ii) increased autophagy flux and co-localization of p62—an autophagy adaptor protein—with ABCD3 in the presence of the autophagy inhibitor chloroquine. However, the levels of cholesterol and plasmalogens did not recover despite the restoration of peroxisome abundance following chloroquine treatment. Thus, PEX16 is indispensable for maintaining peroxisome homeostasis by regulating not only the commonly known biogenesis pathway but also the autophagic degradation of peroxisomes.

## 1. Introduction

Peroxisomes are single membrane bound organelles that exist in almost all eukaryotic cells [1]. They carry out a variety of cellular metabolic functions, such as oxidation of very long chain fatty acids (VLCFAs) and branched chain fatty acids, synthesis of plasmalogens, and removal of reactive oxygen species (ROS) [2,3]. Because of their importance in cellular metabolism, peroxisome abundance is tightly regulated by maintaining a balance between peroxisome biogenesis and degradation, which depends on the metabolic requirements and physiological states of the cells [4,5].

Peroxisome biogenesis occurs through two pathways—de novo biogenesis and fission of pre-existing peroxisomes—which are orchestrated by proteins belonging to the family of peroxins (PEX-proteins) [6]. PEX19 is a cytosolic chaperone that transports the peroxisomal membrane proteins (PMPs) to the peroxisomal membrane, where the PMPs dock with a complex containing PEX16 and PEX3 [7,8]. PEX16 is an integral membrane protein that acts as a receptor of PEX3 at both the endoplasmic reticulum (ER) [9] and peroxisome [10] to facilitate PMP import. In addition, peroxisomes can undergo growth and division from pre-existing peroxisomes; the elongation and fission of peroxisomes is regulated primarily by the PEX11 and dynamin-related protein 1 (DRP1) [11]. Abnormal peroxisome assembly and impaired peroxisomal function have been shown to cause a spectrum of autosomal recessive metabolic disorders, referred to as peroxisomal biogenesis disorders [12].

Peroxisomes are dynamic organelles that undergo routine turnover for organelle quality control, as well as degradation under environmental changes, including nutrient deprivation, hypoxia, and high ROS levels [4,13]. Several findings suggest that peroxins involved in peroxisome biogenesis may also contribute to pexophagy. Overexpression of membrane peroxin PEX3 has been shown to induce peroxisome ubiquitination, leading to pexophagy activation [14]. Defects in the peroxisome matrix import machinery have also been shown to act as a signal to trigger pexophagy [4,13]. The loss of AAA-ATPase complex (AAA-complex) function in cells results in the accumulation of ubiquitinated PEX5 on the peroxisomal membrane, which activates pexophagy. Genetic or pharmacological inhibition of autophagy rescues the number and function of peroxisomes [15].

Although membrane peroxins including PEX3, PEX16, and PEX19 have been associated with peroxisome biogenesis, the knockdown effect of these peroxins on peroxisome abundance remains controversial [14,16,17,18]. Therefore, the present study aimed to investigate whether the deficiency of PEX16 might mediate peroxisome loss through either autophagy-dependent or -independent mechanisms. Here, we demonstrate that PEX16, but not PEX3 and PEX19, plays a role in modulating not only biogenesis but also autophagic degradation of peroxisomes

## 2. Results

### 2.1. Knockdown of PEX3, PEX16, and PEX19 Decreases Peroxisome Abundance in RPE-1 Cells

**Figure 1:**
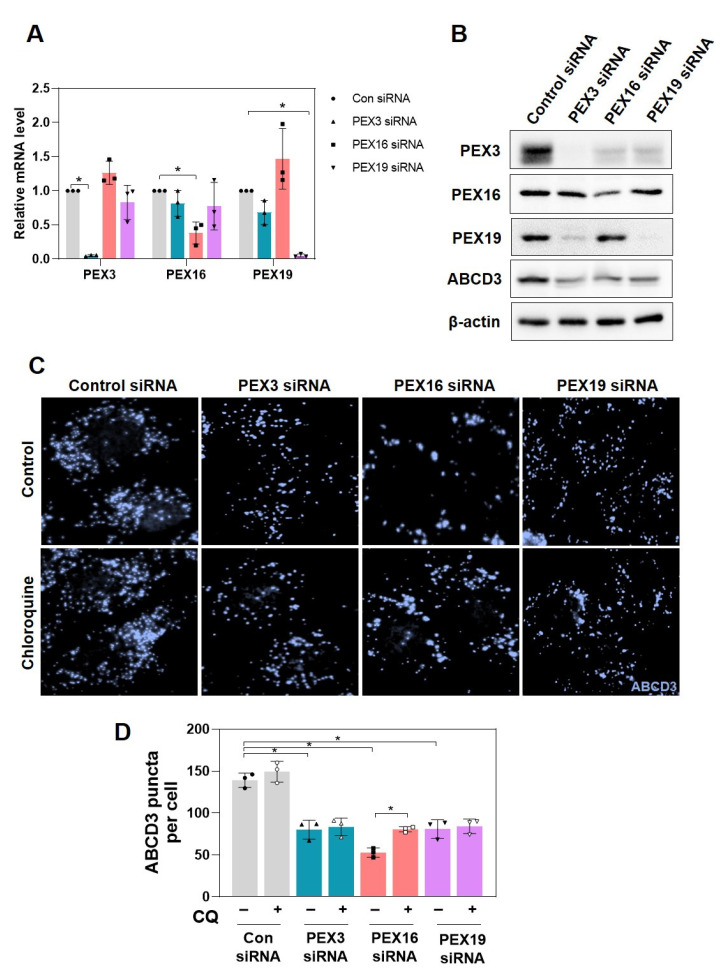
Knockdown of PEX3, PEX16, and PEX19 decreases peroxisome abundance in RPE-1 cells. RPE-1 cells were transfected with siRNA of PEX3, PEX16, and PEX19 for 72 h. The transfection efficiency was evaluated by measuring (**A**) the relative mRNA levels with RT-qPCR and (**B**) the protein expression by immunoblotting. (**C**) Cells were treated with siRNA of PEX3, PEX16, and PEX19 for 48 h and treated with 5 μM chloroquine (CQ) for an additional 24 h. Cells were subjected to immunofluorescence to examine the expression of ABCD3 puncta. (**D**) Quantification of ABCD3 puncta represented the number of peroxisomes per cell. Data are expressed as means ± S.D. (*n* = 3, independent experiments), * *p* < 0.05.

We first examined whether knockdown of peroxins involved in de novo peroxisome biogenesis affected peroxisome abundance in retinal pigment epithelial-1 (RPE-1) cells. After treatment with siRNAs of PEX3, PEX16, and PEX19, the transfection efficiency was demonstrated by decreases in relative mRNA and protein expression levels of these peroxins as compared to control scramble siRNA (Figure 1A,B). Knockdown of peroxins including PEX3, PEX16, and PEX19 significantly decreased peroxisome abundance, indicated by reductions in ATP-binding cassette sub-family D member 3 (ABCD3) protein expression (Figure 1B) and the number of ABCD3 puncta in RPE-1 cells (Figure 1C). In addition, cells with PEX3, PEX16, and PEX19 deficiency were further treated with chloroquine, a well-known pharmacological inhibitor of autophagy. Chloroquine treatment partially recovered the peroxisome abundance in PEX16 knockdown, but not in either PEX3 or PEX19 knockdown (Figure 1C,D). This observation suggests that peroxisome loss under PEX16 deficiency may result from the autophagic degradation of peroxisomes.

### 2.2. Autophagy Mediates the Loss of Peroxisomes in RPE-1 Cells with PEX16 Knockdown

**Figure 2:**
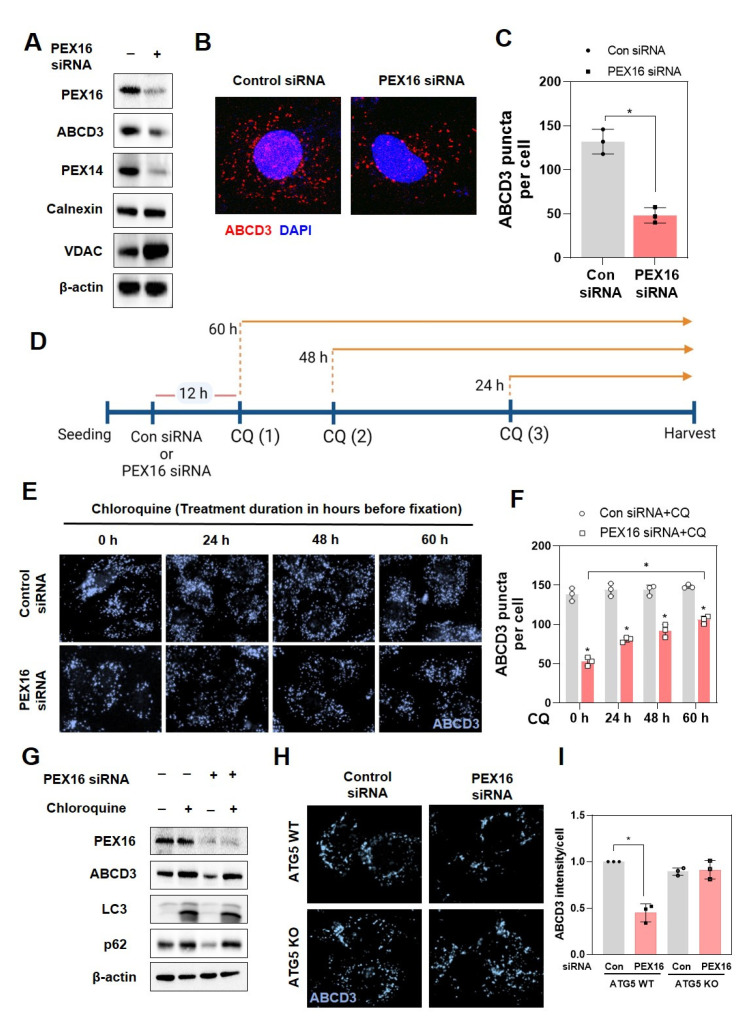
Pexophagy mediates the loss of peroxisomes in RPE-1 cells with PEX16 knockdown. (**A**) Western blot analysis was used on RPE-1 cell lysates to check the protein expression of PEX16, ABCD3, PEX14, Calnexin, and VDAC, using β-actin as an internal control. (**B**) Cells were treated with PEX16 siRNA for 72 h, immunostained with ABCD3, and (**C**) the number of peroxisomes per cell was quantified. Data are expressed as means ± S.D. (*n* = 3, independent experiments), * *p* < 0.05. (**D**) Cells were transfected with PEX16 siRNA, and then treated with chloroquine (CQ) at different time intervals as indicated. (**E**) Then, cells were subjected to immunofluorescence with ABCD3 antibody. (**F**) The number of ABCD3 puncta per cell was quantified. All data are presented as means ± S.D. (*n* = 3, independent experiments), * *p* < 0.05. (**G**) Cells were treated with either control siRNA or PEX16 siRNA for 12 h, incubated with 5 μM CQ for 60 h, and the protein expression of PEX16, LC3, and p62 was examined with western blotting. (**H**) Wild type and ATG5 null MEF cells were treated with PEX16 siRNA for 72 h, and then subjected to immunofluorescence analysis with ABCD3 antibody. (**I**) Quantification of ABCD3 intensity from (**H**). All data are presented as means ± S.D. (*n* = 3, independent experiments), * *p* < 0.05.

We further investigated whether autophagy mediated peroxisome loss in PEX16 knockdown cells. Protein expression of ABCD3 and PEX14, which represent peroxisomal membrane markers [1,19], was decreased in RPE-1 cells with PEX16 knockdown (Figure 2A). Decreased peroxisome abundance was also suggested by a reduction in ABCD3 puncta per cell, as detected by immunofluorescence (Figure 2B,C). Moreover, calnexin, an endoplasmic reticulum protein, and VDAC, a mitochondrial protein, did not decrease in RPE-1 cells with PEX16 knockdown (Figure 2A).

Since the above results showed that chloroquine treatment only partially rescued the peroxisome abundance in the presence of PEX16 siRNA for 48 h (Figure 1C), we wanted to examine the effects of autophagy inhibition in a time-dependent manner. Cells were treated with chloroquine at 12 h, 24 h, and 48 h after transfection with either control or PEX16 siRNA, and then further maintained for three different durations of treatment: 60 h, 48 h, and 12 h, respectively (Figure 2D). As expected, the number of peroxisomes, represented as ABCD3 puncta, was recovered by the addition of chloroquine in a time-dependent manner in cells with PEX16 knockdown. Control cells treated with chloroquine did not exhibit any change in peroxisome number regardless of the duration of treatment (Figure 2E,F).

In addition to the elevation of ABCD3-positive fluorescence, chloroquine treatment in PEX16 knockdown increased ABCD3 protein (a marker of peroxisome abundance) and inhibited autophagy, represented by a decrease in the LC3 II/I ratio and an increase in p62 protein (Figure 2G). Supporting these results, PEX16 knockdown in mouse embryonic fibroblast (MEF) cells demonstrated a reduction in ABCD3 protein expression as compared to control cells, which was abrogated in autophagy-deficient autophagy-related gene 5 (ATG5) knockout MEF cells (Figure 2H,I). Collectively, these results emphasize that the autophagy process is required for the degradation of peroxisomes during PEX16 knockdown.

### 2.3. p62 Mediates Pexophagy in RPE-1 Cells with PEX16 Knockdown

**Figure 3:**
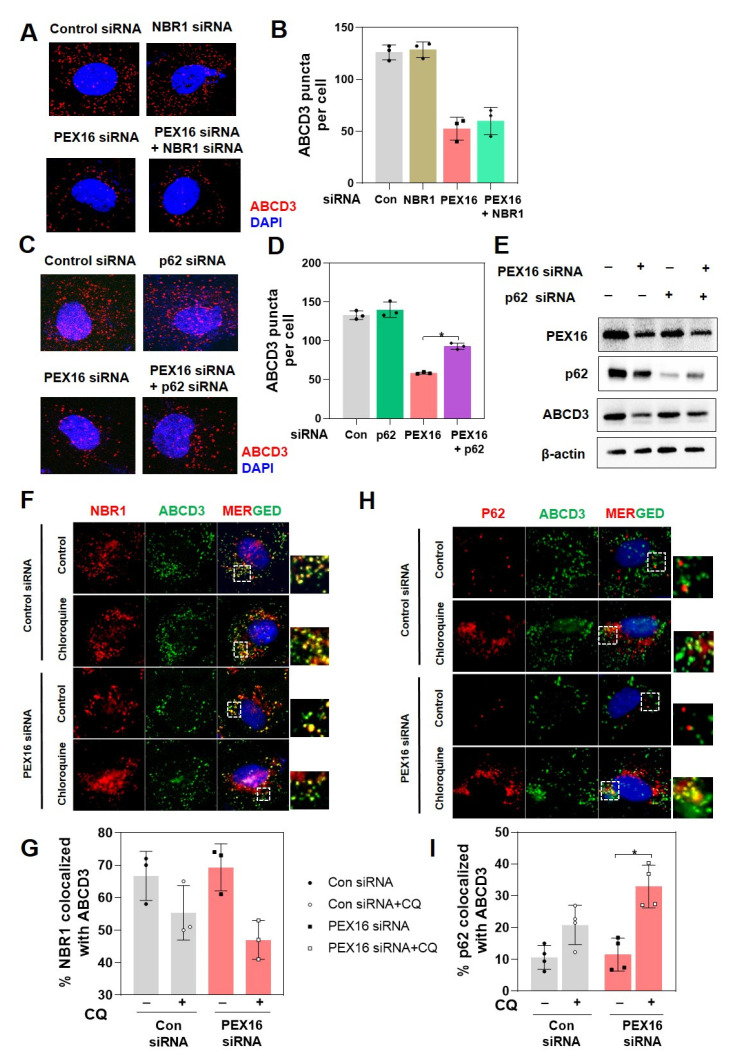
p62 mediates pexophagy in RPE-1 cells with PEX16 knockdown. (**A**) Cells were co-treated with siRNA of NBR1 and PEX16, followed by immunostaining for ABCD3. (**B**) Quantification of ABCD3 puncta per cell from (**A**). (**C**) Cells were co-treated with p62 and PEX16 siRNA and immunostained for ABCD3. (**D**) Quantification of ABCD3 puncta per cell (**C**). Data are expressed as mean ± S.D. (*n* = 3, independent experiments), * *p* < 0.05. (**E**) Cell lysates were also analyzed by western blot to determine the protein expression of PEX16, p62, and ABCD3. Cells were treated with either control siRNA or PEX16 siRNA for 12 h and incubated with 5 μM chloroquine (CQ) for 60 h. (**F**) Co-localization of ABCD3 and NBR1 was analyzed by co-immunostaining. (**G**) Quantification of NBR1/ABCD3 co-localization. (**H**) Co-localization of ABCD3 and p62 was analyzed by co-immunostaining. (**I**) Quantification of p62/ABCD3 co-localization. All data are presented as means ± S.D. (*n* = 3–4, independent experiments), * *p* < 0.05.

To verify that pexophagy contributed to the loss of peroxisomes in PEX16 knockdown cells, cells were co-transfected with siRNA of p62 or neighbor of BRCA1 gene 1 (NBR1). p62 and NBR1 have been shown to act as autophagy receptors that contain LC3- and ubiquitin-binding domains [20,21]. The transfection efficiency of the NBR1 siRNA was confirmed (Figure S1A). We showed that double knockdown of PEX16 and NBR1 did not restore peroxisome abundance (Figure 3A,B). However, double knockdown of PEX16 and p62 significantly recovered peroxisome abundance, as demonstrated by an increase in ABCD3 expression detected by immunofluorescence (Figure 3C,D) and immunoblotting (Figure 3E), suggesting that p62 could be a possible pexophagy receptor. To confirm this, we examined the co-localization of these factors with peroxisome membrane proteins. The levels of p62 co-localized with ABCD3 were significantly increased when cells with PEX16 knockdown were treated with chloroquine. However, NBR1 was already localized in the peroxisomes under basal conditions, which was similar to a previous study [22]. NBR1 co-localized with ABCD3 did not significantly change under PEX16 knockdown and chloroquine treatment (Figure 3F–I). Thus, our results suggest that PEX16 knockdown results in autophagic degradation of cells, which is mediated by p62 as an autophagy receptor.

### 2.4. Peroxisome Function Is Not Recovered by Chloroquine in Cells with PEX16 Knockdown

**Figure 4:**
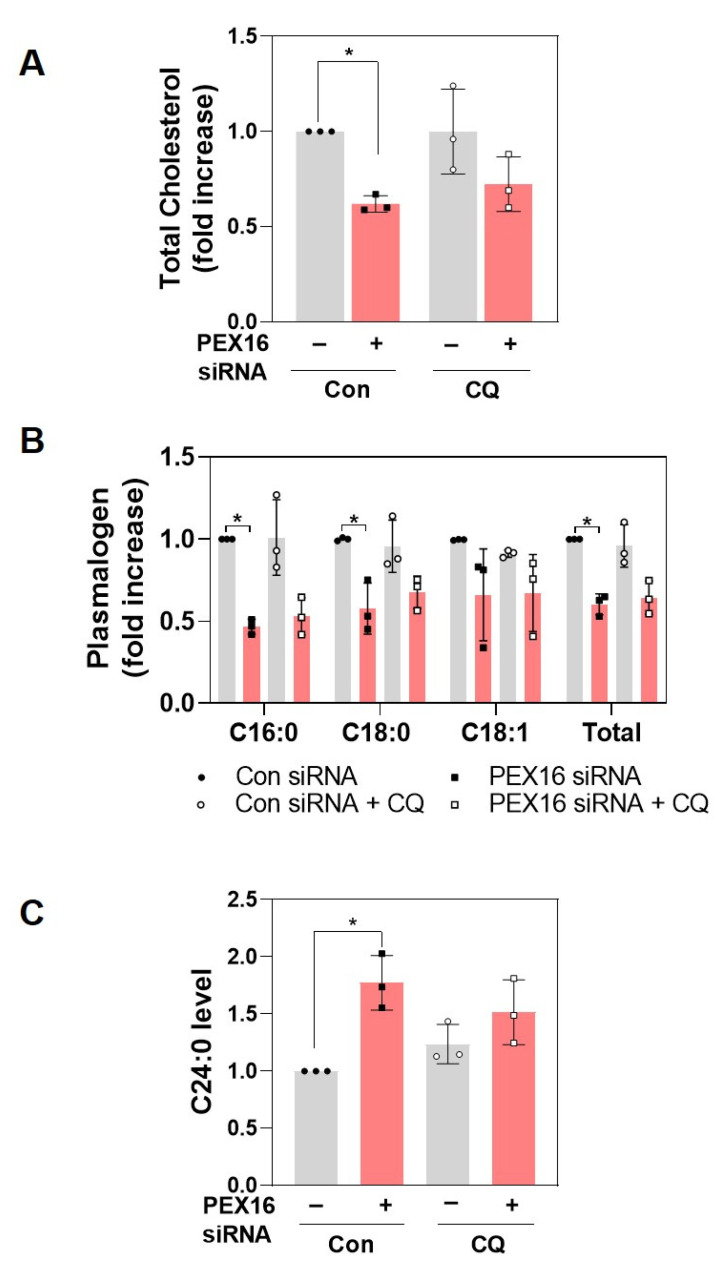
Peroxisome function is not recovered by chloroquine in cells with PEX16 knockdown. RPE-1 cells were treated with siRNA of either control or PEX16 for 12 h, followed by incubation with 5 μM chloroquine for 60 h. Under this condition, (**A**) the levels of cholesterol and (**B**) plasmalogens were measured. (**C**) VLCFA (C24:0) level was measured. All data are presented as means ± S.D. (*n* = 3, independent experiments), * *p* < 0.05.

Peroxisomes play roles in a variety of metabolic functions, such as fatty acid oxidation and lipid synthesis [2,3]. The biosynthesis of plasmalogen is initiated in peroxisomes and completed in the ER [23], while several reports suggest the role of peroxisomes in cholesterol biosynthesis remains controversial [24,25,26]. Our data showed that the levels of total cholesterol and plasmalogen were significantly reduced in PEX16 knockdown cells, and they were not restored by chloroquine treatment (Figure 4A,B). Furthermore, a study has reported that chloroquine treatment in PEX1-mutated fibroblast decreased VLCFA accumulation [15]. However, the accumulation of VLCFA due to PEX16 knockdown was not reduced by chloroquine treatment in RPE-1 cells (Figure 4C). Thus, the results shown in Figure 4 suggest that the inhibition of pexophagy in PEX16 knockdown cells does not restore peroxisome functions.

## 3. Discussion

Along with PEX3 and PEX19, PEX16 is classically recognized as a peroxin that plays a role in the initial step of peroxisome biogenesis [1]. We provide a novel finding that PEX16 is involved in the maintenance of peroxisome abundance by modulating the autophagic degradation of peroxisomes in RPE-1 cells. Autophagy inhibition by chloroquine significantly restored peroxisome abundance following PEX16 knockdown. We consistently observed knockdown of PEX16 using Santa Cruz siPEX16 (Santa Cruz Biotechnology, Dallas, TX, USA) reduced peroxisome abundance and inhibition of autophagy with chloroquine restored peroxisome abundance (Figure S2).

The present study demonstrated that PEX16 knockdown decreased peroxisome abundance and function, represented by reductions in the protein expression of ABCD3 and the levels of cholesterol and plasmalogens, respectively. Supporting our findings, PEX16 silencing with shRNA in 3T3-L1 fibroblasts reduced peroxisome number and decreased protein and gene expression involved in peroxisome biogenesis and function [17]. However, another study showed no statistically significant differences in the number of catalase puncta in HeLa cells transfected with PEX16 siRNA for two days [15]. Different experimental conditions could contribute to the discrepancies found in these independent studies.

In the present study, the activation of pexophagy under PEX16 knockdown was supported by (i) abrogated peroxisome loss under autophagy deficiency in ATG5 knockout cells, and (ii) inhibited autophagy flux under chloroquine treatment, which is indicated by increased co-localization of p62 with ABCD3 and protein expression of p62 and LC3-II. Furthermore, PEX16 knockdown also induced autophagosome formation genes, such as LC3, Beclin1, and GABARAP (Figure S3). However, the mechanism underlying the induction of autophagy at the transcriptional level remains unclear. Further studies are required to determine whether autophagy activation is due to metabolic disturbances following PEX16 knockdown.

Pexophagy encompasses all processes of general autophagy, including binding of ubiquitinated proteins with peroxisomes through autophagy-related receptor proteins, such as p62 and NBR1, formation of autophagosomes, and fusion and degradation of pexophagic cargoes in lysosomes [4,27]. Unlike these widely accepted criteria, our results showed no involvement of NBR1 in pexophagy under PEX16 knockdown by siRNA. Another study has also reported that NBR1 knockdown could not restore peroxisome numbers in hepatoma-derived Huh7 cells under hypoxia-induced peroxisome degradation [28]. However, future studies are necessary to demonstrate how PEX16 interacts with the pexophagy machinery.

Ubiquitinated PEX5 has been shown to act as an important signal to trigger pexophagy in mammalian cells. Excessive ROS levels induced ataxia-telangiectasia mutated (ATM)–PEX5 interactions in peroxisomes, leading to enhanced PEX5 phosphorylation by ATM and ubiquitination by PEX2. This event facilitated p62-mediated pexophagy [29]. Our present study demonstrated that PEX16 knockdown did not increase ROS levels (Figure S1B) or PEX5 ubiquitination, the classical cues of pexophagy. PEX5 ubiquitination was not observed after the inhibition of autophagy by chloroquine following PEX16 knockdown (Figure S1C). However, the ablation of p62 remarkably restored peroxisome abundance in cells with PEX16 knockdown. An increase in ABCD3 protein expression was consistently observed in human embryonic kidney (HEK) 293T cells with PEX16 and p62 knockdown (Figure S4). Therefore, these results suggest the existence of distinct and unidentified ubiquitinated-proteins on the peroxisome membrane that can be recognized by adaptor proteins and cue pexophagy induction. In addition, a study has shown that LC3-II can compete with PEX5 for binding to the N-terminal region of PEX14 and further facilitate pexophagy [30]. This mechanism highlights that peroxisomes may be marked for pexophagy depending on the abundance of a certain peroxin, instead of ubiquitin.

In the present study, we showed that autophagy inhibition in PEX16 knockdown could not fully recover peroxisome abundance. Although both peroxisome fission and pexophagy are critical in maintaining peroxisome abundance [11], whether peroxisome fission was affected during PEX16 knockdown remains unclear. In addition, we showed that even though siRNA of PEX16 suppressed the expression level of PEX16, chloroquine treatment still restored the peroxisome abundance while peroxisome functions were not rescued. We speculated that the restored peroxisomes may be dysfunctional since the loss of PEX16 possibly affects the transportation of unknown proteins. However, the enigmatic mechanism requires further investigations.

Overall, we demonstrated that PEX16 knockdown induces autophagic degradation of peroxisomes, which require p62 proteins. We believe that the present study can be used as a starting point to explore the role of PEX16 in the regulation of pexophagy in other mammalian cells and in various tissues.

## 4. Materials and Methods

### 4.1. Reagents and Antibodies

Reagents and antibodies used were as follows: anti-Calnexin (#ab22595, Abcam, Cambridge, MA, USA), anti-LC3 (#L8918, Sigma-Aldrich, St. Louis, MO, USA), anti-NBR1 (#16004-1-AP, Proteintech, Rosemont, IL, USA), anti-PEX3 (#247042, Abcam), anti-PEX14 (#sc-23197, Santa Cruz Biotechnology, Dallas, TX, USA), anti-PEX16 (#14816-1-AP, Proteintech, Rosemont, IL, USA), anti-PEX19 (#PA5-22129, Invitrogen, Carlsbad, CA, USA), anti-ABCD3 (#ab3421, Abcam, Cambridge, MA, USA and #SAB4200181, Sigma-Aldrich, St. Louis, MO, USA), anti-SQSTM1/p62 (#H00008878-M01, Abnova, Taipei, Taiwan), anti-VDAC (#55259-1-AP, Proteintech, Rosemont, IL, USA), anti-β-actin (#sc-47778, Santa Cruz Biotechnology, Dallas, TX, USA), and chloroquine (#C6628, Sigma-Aldrich, St. Louis, MO, USA).

### 4.2. Cell Culture

Human retinal pigmented epithelial (RPE)-1 cells (a gift from Dr. Joon Kim, KAIST, Korea) and mouse embryonic fibroblast (MEF) cells were maintained in Dulbecco’s modified Eagle medium (DMEM, Gibco-BRL, Grand Island, NY, USA) supplemented with 10% fetal bovine serum (Gibco-BRL, Grand Island, NY, USA), 100 IU/mL penicillin (Invitrogen, Carlsbad, CA, USA), and 100 μg/mL streptomycin (Invitrogen, Carlsbad, CA, USA) at 37 °C and 5% CO_2_ in humidified air.

### 4.3. siRNA Transfection

PEX knockdown was performed by siRNA transfection with Lipofectamine RNAiMAX (#13778-150, Invitrogen, Carlsbad, CA, USA) according to the manufacturer’s instructions. Cells were harvested 72 h after siRNA transfection. siRNAs directed against PEX3 (5′-CUGUAUGCUGGUUGUUCUU-3′), PEX16 (5′-AGCAGCAUCACGAGGAGCU-3′), PEX19 (5′-GAGAUCGCCAGGAGACACU-3′), and SQSTM1/p62 (5′-CAGACUACGACUUGUGUAG-3′) were custom synthesized by Bioneer (Daejeon, South Korea). NBR1 siRNAs (#sc-94187 [31,32]) were purchased from Santa Cruz Biotechnology (Dallas, TX, USA).

### 4.4. Western Blot Analysis

Cultured cells were harvested from the culture dish and centrifuged at 5000 rpm for 5 min at 4 °C. The pellets obtained were lysed on ice with radioimmunoprecipitation assay buffer (20 mM HEPES pH 7.5, 150 mM sodium chloride, 1% Triton X-100, 1% sodium deoxycholate, and 1 mM EDTA) mixed with protease and phosphatase inhibitor cocktail solution (GenDEPOT, Barker, TX, USA), and lysates were centrifuged at 14,000 rpm for 10 min at 4 °C. SDS loading buffer was added to the supernatant and denatured at 97 °C for 10 min. Protein lysates were subjected to SDS-PAGE. Protein expression levels were quantified using Image Lab 6.1 from Bio-Rad (Hercules, CA, USA). β-actin was used as a protein-loading control.

### 4.5. RNA Isolation and Real-Time qPCR Analysis

Total RNA was extracted using TRIzol reagent (#15596018, Invitrogen, Carlsbad, CA, USA) according to the manufacturer’s instructions. A reverse transcription kit (#04379012001, Roche, Mannheim, Germany) was used to transcribe the cDNA. Real-time qPCR was performed with cDNA as a template using a Light Cycler system with FastStart DNA Master SYBR Green (#06402712001, Roche, Mannheim, Germany). The 36B4 gene was used as an internal control, and the primer sequences used in the present study are listed in Table A1.

### 4.6. Measurement of Total Cholesterol

Total cholesterol was measured using a colorimetric assay kit II (#K623, BioVision, Milpitas, CA, USA), according to the manufacturer’s instructions.

### 4.7. Immunofluorescence Analysis

Cells grown on coverslips were fixed with 4% paraformaldehyde (#HT5014, Sigma-Aldrich, St. Louis, MO, USA) for 20 min at room temperature, permeabilized using 0.5% Triton X-100 in PBS for 10 min, and followed by blocking with 3% bovine serum albumin for 1 h at room temperature. Following overnight incubation with primary antibodies in 3% bovine serum albumin at 4 °C, cells were rinsed with PBS and labeled with fluorescent Alexa Fluor 488- and Alexa Fluor 568-conjugated secondary antibodies (molecular probes) (1:500) in the dark for 1 h at room temperature. Cells were washed twice with PBS and incubated with 10 mM 4,6-diamidino-2-phenylindole (DAPI) in PBS at room temperature for 5 min. Coverslips were mounted on slides and immunofluorescence was examined with either an Olympus FV1000 confocal laser scanning microscope or an IX71 fluorescence microscope (Olympus, Tokyo, Japan).

The co-localization of p62 or NBR1 with ABCD3 was measured using ImageJ software based on Manders’ overlap coefficient (OC) of co-localization, which varies from zero to one. A value of zero corresponds to non-overlapping images, whereas a value of one reflects 100% co-localization between the images being analyzed.

### 4.8. Lipid Extraction and Gas Chromatography Mass Spectrometry (GC-MS) Analysis

Cells were harvested and centrifuged at 5000 rpm for 5 min at 4 °C. Cell pellets were homogenized in PBS, and the protein concentration was measured using the Bradford assay. Homogenates were added to 1.9 mL extraction solution (a chloroform:methanol volumetric ratio of 1:2) and 40 ng C23:0 as an internal standard, and then vortexed for 2 min. Samples were then added to chloroform (625 μL) and vortexed for 30 s, followed by rinsing with deionized water (625 μL) for 30 s. Then, samples were centrifuged at 3260 rpm for 10 min, and the lower phase (organic) layers were collected and transferred to glass tubes. The extracts were dried under a stream of nitrogen at 40 °C. After cooling, the samples were subsequently dissolved in toluene (200 μL) and mixed with methanol (1.5 mL) and acid chloride (300 μL; 8%) for the conversion of lipid to fatty acid methyl esters (FAMEs). Samples were then cooled and mixed with hexane (1 mL) and deionized water (1 mL). The hexane layers were collected and transferred to new tubes, and extraction with hexane was repeated two more times to optimize the collection of FAMEs. Finally, the hexane layer-pooled FAMEs were dried under nitrogen, dissolved in 100% hexane (100 μL), and transferred to a GC vial for analysis with GCMS-QP2020 (Shimadzu, Kyoto, Japan).

### 4.9. Statistical Analysis

Quantitative data were represented as means ± SD from at least three independent experiments, and statistical significance was set at a * *p*-value < 0.05. Based on the design of the experiment, statistical analysis was performed using either a two-tailed Student’s *t*-test or one-way analysis of variance.

## Data Availability

Not applicable.

## Supplementary Materials

The following are available online at https://www.mdpi.com/article/10.3390/ijms22157989/s1, Figure S1: Pexophagy-induced by PEX16 knockdown is not associated to ROS and PEX5 ubiquitination. Figure S2: Inhibition of autophagy restores peroxisome abundance in RPE-1 cells with PEX16 knockdown. Figure S3: The expression of autophagy-related genes in RPE-1 cells with PEX16 knockdown. Figure S4: ABCD3 expression is recovered in HEK293T cells with PEX16 and p62 knockdown.

## Appendix A

**Table A1:** Primer sequences used in the present study.

Genes	Forward	Reverse
36B4	TGCATCAGTACCCCATTCTATCA	AAGGTGTAATCCGTCTCCACAGA
PEX3	TCTGGGGAAATATGGACAGAA	TCGTGCTTGGGCAATGTAT
PEX16	CAAGGTGTGGGGTGAAGTG	TCCGCAGTACAGCCTTGG
PEX19	TGAGGAAGGCTGTAGTGTCG	AATCATCAAGAGCACTTTCCAGA
