# Supplementary material for: Knockdown of PEX16 Induces Autophagic Degradation of Peroxisomes
Source: Int J Mol Sci. 2021 Jul 26;22(15):7989. doi: 10.3390/ijms22157989 (PMC8348608; doi:10.3390/ijms22157989)
Supplement: Supplementary file 1 [file ijms-22-07989-s001.zip › ijms-1269962-supplementary.pdf]

SUPPLEMENTARY MATERIALS

Knockdown of PEX16 Induces Autophagic Degradation of Peroxisomes

Xiaofan Wei †, Yunash Maharjan †, Debra Dorotea †, Raghbendra Kumar Dutta, Donghyun Kim, Hyunsoo Kim, Yizhu Mu, Channy Park and Raekil Park *

Department of Biomedical Science & Engineering, Gwangju Institute of Science and Technology,
Gwangju 61005, Korea; weixiaofan1@gist.ac.kr (X.W.); yunas_rose@yahoo.com (Y.M.); flodebra@gist.ac.kr (D.D.); raghavdutta123@gist.ac.kr (R.K.D.); paul1480@gist.ac.kr (D.K.); ruyhyunsookim@gist.ac.kr (H.K.); yizhumu1202@outlook.com (Y.M.); channy2002@gmail.com (C.P.)

* Correspondence: rkpark@gist.ac.kr; Tel.: +82-(62)-7155361

† Authors contributed equally to this work.

SUPPLEMENTARY METHOD

Measurement of Reactive Oxygen Species (ROS)

Intracellular ROS generation was detected using 2',7'-dichlorofluorescein diacetate (CM-H_2DCFDA) (#C6827, Invitrogen, Eugene, OR, USA). RPE-1 cells were treated with *PEX16* siRNA for 72 h. DCFDA (1 μM) was added to the cells, followed by incubation for 45 min at 37°C in a 5% CO_2 incubator. Cells were then fixed, and the fluorescence intensity was examined with an IX71 fluorescence microscope (Olympus, Tokyo, Japan).

Immunoprecipitation

Immunoprecipitation was performed using Pierce Classic IP Kit (#26146, Pierce Biotechnology, Rockford, IL, USA) according to the manufacturer's instructions.

SUPPLEMENTARY TABLE

Table S1. Primer sequences used in the present study

Genes	Forward	Reverse
36B4	TGCATCAGTACCCCATTCTATCA	AAGGTGTAATCCGTCTCCACAGA
BECLIN1	AGGATGGTGTCTCTCGAAGATT	GATCAGAGTGAAGCTATTAGCATCTTC
GABARAP	GCGAGAAAATCCGAAAGAAA	AGATCAGAAGGCACCAGGTATT
LC3B	CGCACCTTCGAACAAAGAG	CTCACCCTTGTATCGTTCTATTATCA
PEX16-1	CTGGTGTACTCTGCCTCTAAC	CATCTCCATGAACACCTCCA
PEX16-2	ACCATCCTGCTGCTCTACTA	TAATCCATGAGCGGCCTTG

SUPPLEMENTARY FIGURES

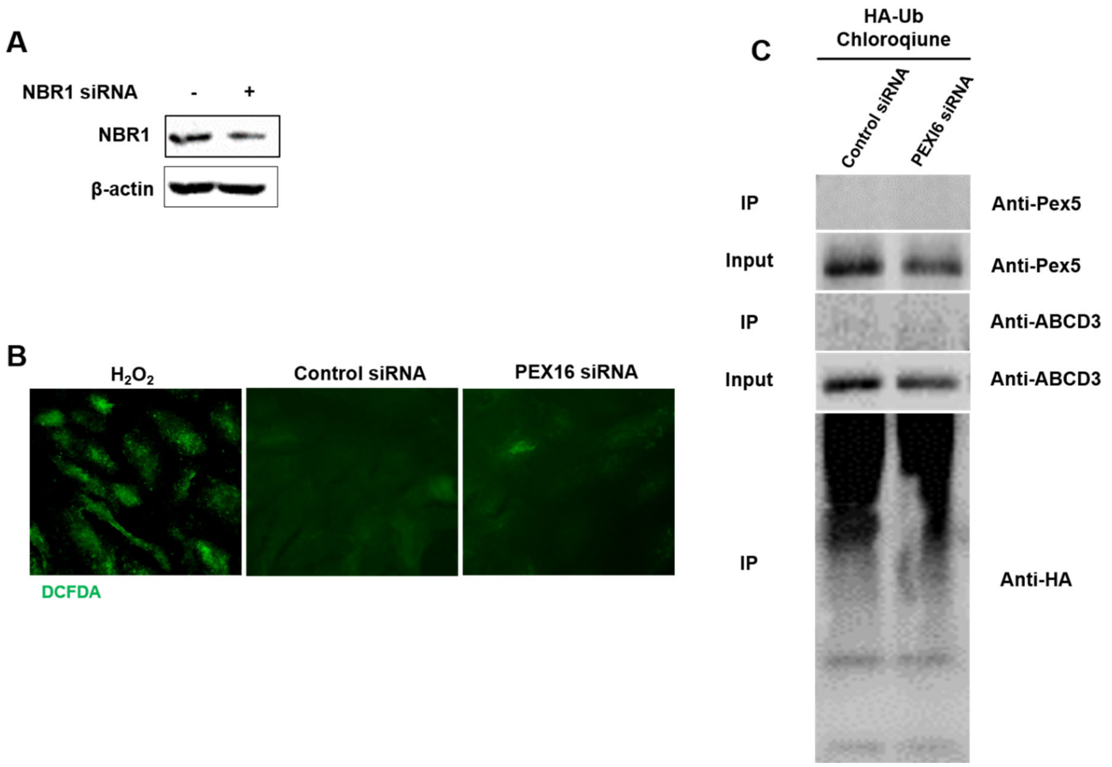

Figure S1. Pexophagy-induced by *PEX16* knockdown is not associated to ROS and PEX5 ubiquitination. **(A)** The immunoblot, confirming the transfection efficiency of NBR1. **(B)** The cellular ROS was detected using DCFDA. **(C)** RPE-1 cells were treated with *PEX16* siRNA for 48 h, transfected with HA-Ubiquitin plasmid in the presence of chloroquine for 24 h, then harvested and subjected to immunoprecipitation.

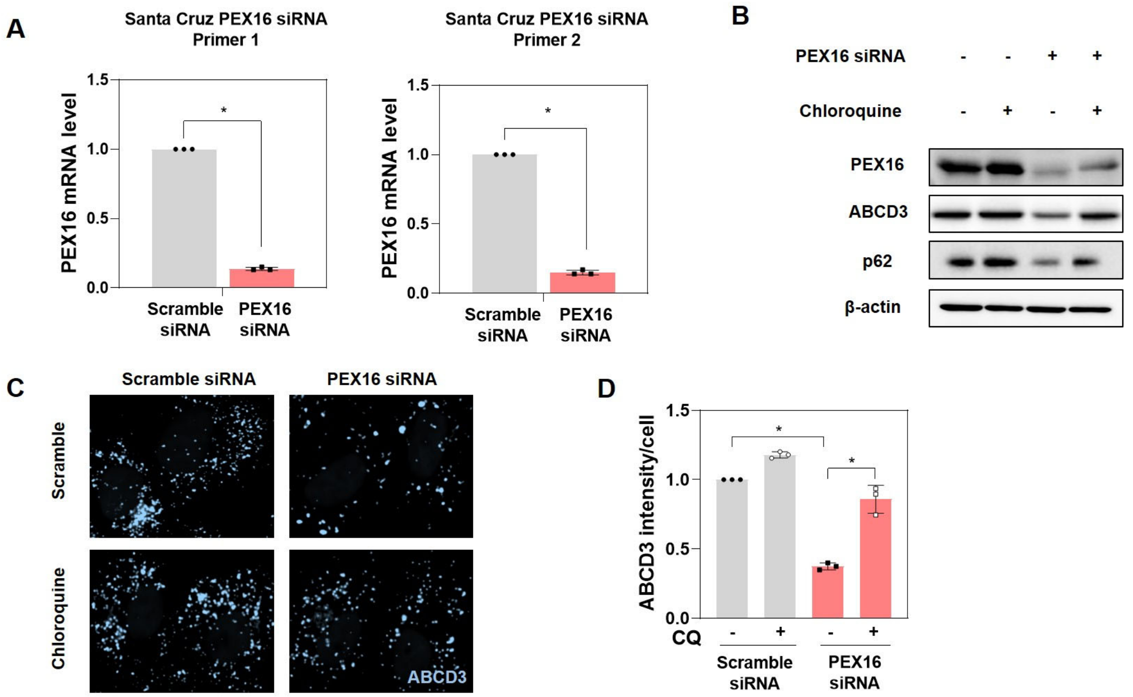

Figure S2. Inhibition of autophagy restores peroxisome abundance in RPE-1 cells with *PEX16* knockdown. **(A)** RPE-1 cells were transfected with siRNA of *PEX16* (Santa Cruz, #sc-96993) for 72 h. The transfection efficiency was evaluated by measuring the *PEX16* mRNA level with RT-qPCR. **(B)** Cells were treated with either Scramble siRNA or *PEX16* siRNA for 12 h, followed by incubation with 5 μM chloroquine (CQ) for 60 h. The protein expression of PEX16, ABCD3, and p62 was examined with immunoblotting. **(C)** The ABCD3 expression was also analyzed using immunofluorescence staining. **(D)** Quantification of ABCD3 intensity from (C). All data are presented as means ± S.D. (n = 3, independent experiments), *$p < 0.05$.

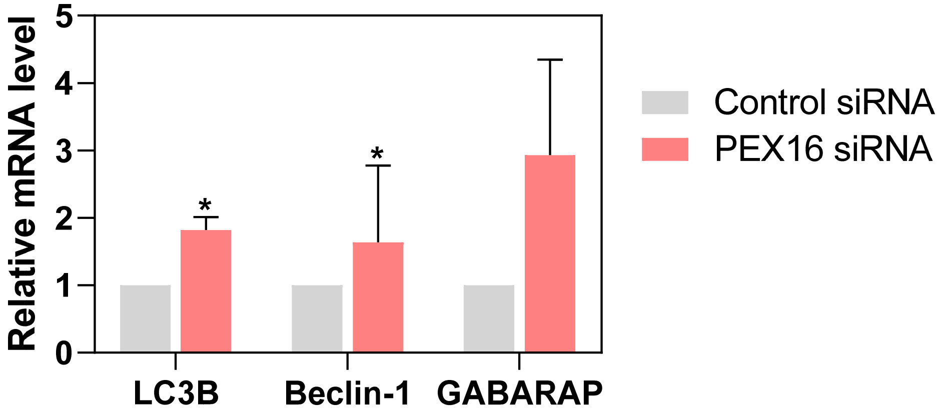

Figure S3. The expression of autophagy-related genes in RPE-1 cells with *PEX16* knockdown. RPE-1 cells were treated with siRNA of either control or *PEX16* for 72 h, and the transcription level of autophagy related genes was analyzed with RT-qPCR.

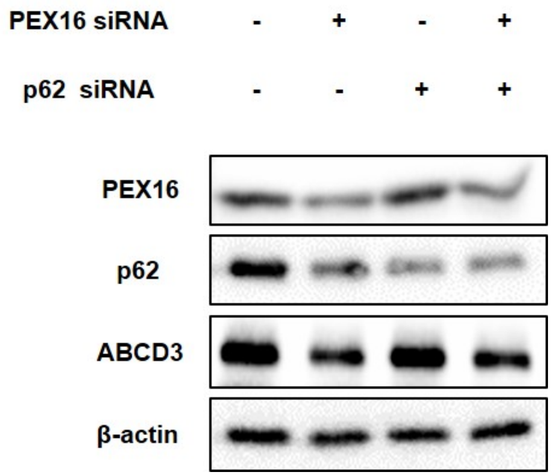

Figure S4. ABCD3 expression is recovered in HEK293T cells with *PEX16* and *p62* knockdown**.** HEK293T cells were co-transfected with siRNA of p62 and PEX16, and the protein lysate was analyzed by immunoblotting to check the expression of PEX16, p62, and ABCD3.
